# Nurse staffing issues; just the tip of the iceberg

**DOI:** 10.1186/1472-6963-14-S2-P136

**Published:** 2014-07-07

**Authors:** Catharina J van Oostveen, Elke Mathijssen, Hester Vermeulen

**Affiliations:** 1 Department of Quality Assurance & Process Innovation, Academic Medical Center, Amsterdam, The Netherlands; 2 Department of Surgery, Academic Medical Center, Amsterdam, The Netherlands; 3 Clinical Health Sciences, Faculty of Medicine, Utrecht University, Utrecht, The Netherlands; 4 Amsterdam School of Health Professions, University of Amsterdam, Amsterdam, The Netherlands

## Background

In response to an increasing health care demand due to the rising age and complexity of patients, hospital boards have implemented nurse-to-patient-ratio’s and patient classification systems. However, on the nursing ward, nurses feel that staffing levels have become critically low, which jeopardises the quality and safety of their patient care. The aim of this study is to obtain in-depth insight into the perceptions of nurses on the current nurse staffing levels in the Netherlands and the use of nurse-to-patient-ratios and patient classification systems.

## Materials and methods

This qualitative study was undertaken on 24 clinical wards, comprising four specialities (surgery, internal medicine, neurology, obstetrics & gynaecology and paediatrics) in a 1000-bed Dutch university hospital. Four focus groups (n = 44 nurses) and 27 interviews (with 20 head nurses, four nursing directors and three quality advisors) were conducted. Data were collected from September until December 2012.

## Results

Nurse staffing issues appear to be merely the ‘tip of the iceberg’. Below the surface three underlying main themes became clear; nursing behaviour, authority, and autonomy, cross-cut by a single overall theme; nurses’ position. In general, nurses’ behaviour, way of thinking, decision-making, and communicating thoughts or information differs from other disciplines like physicians and quality advisors. This results in a perceived and actual lack of authority and autonomy. This in turn hinders them to plead for adequate nurse staffing in order to achieve the common goal of safe and high-quality patient care. Nurses desired a validated nursing care intensity score as interdisciplinary and objective communication tool that makes nursing care visible and creates possibilities for a better positioning of nurses in hospitals and a further professionalization in terms of enhanced authority and autonomy.

## Conclusions

The subservient position of clinical nurses seems the underlying root cause of nurse staffing problems. It is yet unknown whether an objective nursing care intensity score would truly help them communicate effectively and credibly and subsequently improve their own position.

